# How populations differentiate despite gene flow: sexual and natural selection drive phenotypic divergence within a land fish, the Pacific leaping blenny

**DOI:** 10.1186/1471-2148-14-97

**Published:** 2014-05-06

**Authors:** Courtney L Morgans, Georgina M Cooke, Terry J Ord

**Affiliations:** 1 Evolution and Ecology Research Centre, School of Biological, Earth and Environmental Sciences, University of New South Wales, Kensington, NSW, Australia; 2 The Australian Museum, Ichthyology, Australian Museum Research Institute, 6 College Street, Sydney, NSW, Australia

**Keywords:** Adaptation, Neutral evolution, Secondary sexual trait, Selection trade-off, Static allometry

## Abstract

**Background:**

Divergence between populations in reproductively important features is often vital for speciation. Many studies attempt to identify the cause of population differentiation in phenotype through the study of a specific selection pressure. Holistic studies that consider the interaction of several contrasting forms of selection are more rare. Most studies also fail to consider the history of connectivity among populations and the potential for genetic drift or gene flow to facilitate or limit phenotypic divergence. We examined the interacting effects of natural selection, sexual selection and the history of connectivity on phenotypic differentiation among five populations of the Pacific leaping blenny (*Alticus arnoldorum*), a land fish endemic to the island of Guam.

**Results:**

We found key differences among populations in two male ornaments—the size of a prominent head crest and conspicuousness of a coloured dorsal fin—that reflected a trade-off between the intensity of sexual selection (male biased sex ratios) and natural selection (exposure to predators). This differentiation in ornamentation has occurred despite evidence suggesting extensive gene flow among populations, which implies that the change in ornament expression has been recent (and potentially plastic).

**Conclusions:**

Our study provides an early snapshot of divergence in reproductively important features that, regardless of whether it reflects genetic or plastic changes in phenotype, could ultimately form a reproductive barrier among populations.

## Background

The emergence of phenotypic divergence among populations of the same species, especially in features important for reproduction, is a central concept in speciation [1]. This is because the accumulation of phenotypic changes in different populations is often critical for instigating—and subsequently maintaining—reproductive isolation between populations. There are three possible ways by which phenotypic differentiation might originate among populations: via sexual selection, natural selection, or genetic drift. In the case of sexual selection, there is extensive evidence from numerous taxa that shows sexual selection can elaborate or otherwise increase the magnitude of characteristics that influence an animal’s reproductive success (e.g., [2-5]). Populations that differ in the intensity of sexual selection should subsequently differ in the elaboration or size of characteristics that influence reproduction (e.g., [6,7]). However, natural selection on the same features can have different effects. In particular, sexually selected features are often conspicuous to both prospective mates and predators (e.g., [8]; but see [9]), and differences in predation pressure among populations can subsequently determine the extent to which ornaments can be elaborated by sexual selection [10,11].

In addition to the phenotypic variance caused by sexual and natural selection, populations can diverge from one another through the process of neutral evolution. Here, divergence between populations occurs as the direct accumulation of genetic drift. The extent to which neutral evolution in itself might ultimately instigate reproductive isolation among populations is debatable [12,13]. However, theoretical models and a growing number of empirical studies have begun to link population variation in mating and territorial signals to neutral genetic differentiation among populations [14-20]. At the very least, then, neutral evolution among populations represents an important null model that should be considered in studies of population divergence. Conversely, populations experiencing high gene flow or those that have only recently become isolated from one another are expected to exhibit little phenotypic divergence due to the homogenizing effect of dispersing individuals mating among populations or because mutations have yet to arise in populations to cause phenotypic divergence [21-23]. In this context, populations may appear phenotypically ill suited (maladapted) to their local selection environments unless characteristics are plastic. Without an adequate understanding of the history of connectivity among populations, interpreting an apparent lack of response to selection or a response that might be plastic in origin would subsequently be difficult.

Taken together, sexual selection, natural selection and the degree of genetic differentiation exhibited by populations each have the potential to culminate in (or limit) phenotypic variation among geographically separated populations within the same species. While some of these variables have been examined individually—most notably sexual selection—any study wishing to adequately identify the origin (or lack) of phenotypic divergence among populations needs to consider all three effects simultaneously (e.g. [24]). We did so here for several reproductively important characters in a fish, the Pacific leaping blenny (*Alticus arnoldorum*). This species is a highly social fish that lives its adult life on land and at high densities along the rocky foreshores of the island of Guam. As a model system, the Pacific leaping blenny offers a unique opportunity to study sexual selection and natural selection for the following reasons.

First, despite several key adaptations that allows the Pacific leaping blenny to thrive on land (e.g., see [25,26]) it is still vulnerable to desiccation and is subsequently confined to the intertidal rocks within the splash zone around the island. The fish is further limited by fluctuations in tide level and air temperature: at high and low tides, movement on exposed rocks is inhibited, while at high and low temperatures there is an increased chance of desiccation or it is too cold for terrestrial activity [27]. This brief, variable window of activity means the Pacific leaping blenny is under considerable time pressure to find mates and acquire other resources needed for reproduction (e.g., food, rock holes for nesting). Any shift in the number of mates or competitor density in a population should subsequently translate into large effects on the intensity of sexual selection experienced by fish in that population.

Second, the Pacific leaping blenny is acutely vulnerable to predation on land [28]. Most of its activity is spent out in the open on exposed rocks. Predators include birds, predatory land crabs and lizards (e.g., see [28]). Any differences among populations in predation pressure should also have important consequences for the extent to which populations can express conspicuous ornamentation. In particular, both sexes rely on flashing a large, bright red dorsal fin during courtship and aggressive interactions [27]. This red dorsal fin is highly conspicuous against the rocky background on which fish are typically viewed [28]. The flash of red during dorsal fin displays makes an otherwise cryptically coloured fish highly localisable.

Furthermore, previous research on the Pacific leaping blenny has shown clear differences in the intensity of red in the dorsal fin, the overall size of the dorsal fin and the size of a prominent head crest in males between two populations separated by only a few kilometres [27]. Extensive behavioural observations inferred that population differences in some of these ornamental features were probably attributable to differences in mating competition (e.g., the size of the male dorsal fin and head crest). The potential cause of variation in other characteristics was less obvious (e.g., the colour of the dorsal fin), suggesting some other factor has limited ornament expression in some populations but not others (e.g., predation or genetic drift).

**Figure 1:**
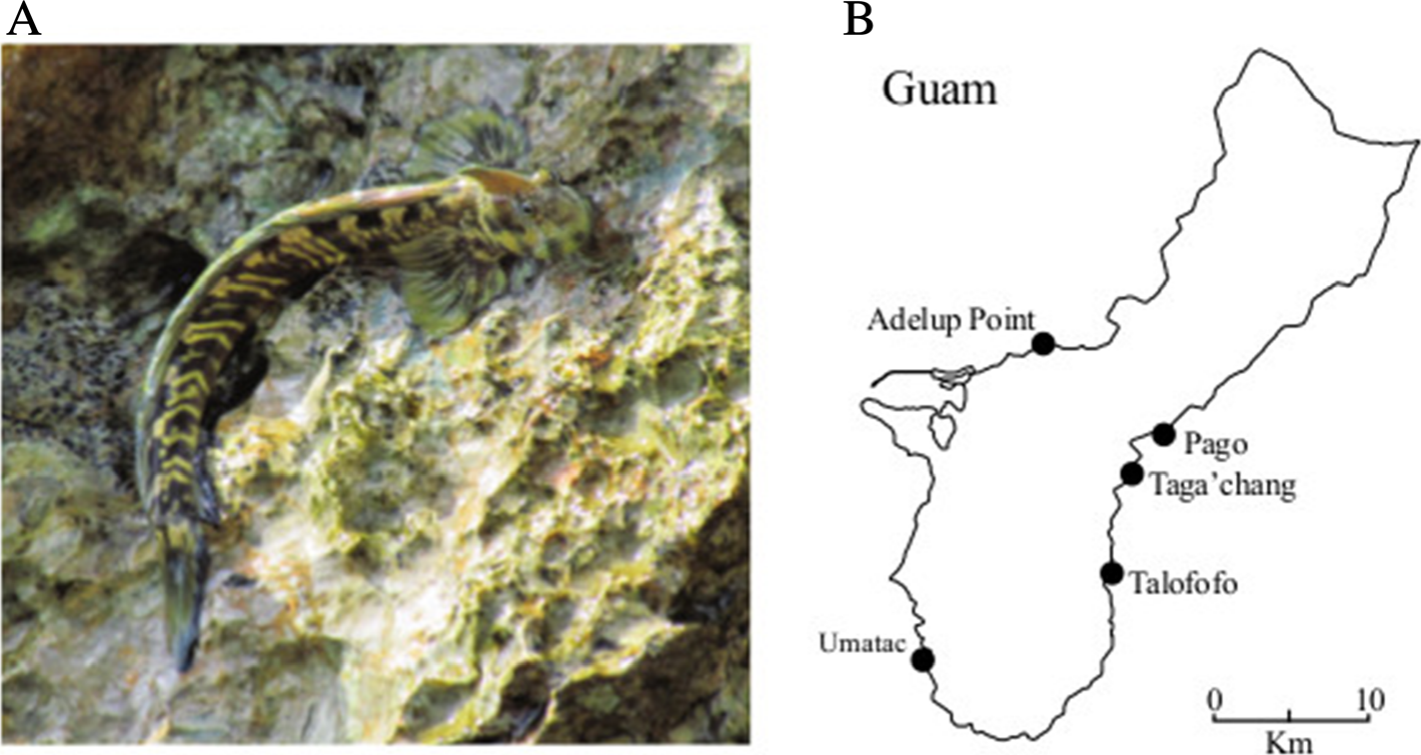
**The Pacific leaping blenny (*A. arnoldorum *) is a marine fish that spends its entire adult life on land.** Adult males have a prominent head crest **(A)**, which is absent in females. Five populations **(B)** at various distances from one another were studied around the island of Guam.

In this study, we intensively surveyed five populations around the island of Guam and measured morphological and ornamental characteristics that included: body size, dorsal fin size, the proportion of the dorsal fin that was conspicuously coloured, the chromatic properties of the dorsal fin (specifically its intensity of red) and the size of the male head crest. We used sex ratio and competitor density to estimate the opportunity for sexual selection. “Flight” distance (an index of predator weariness; [29]) and the number of predator hits to realistic blenny models distributed in the environment [28] were used to estimate the intensity of natural selection from predation on each population. To evaluate the consequences of genetic differentiation (or lack thereof) on phenotypic divergence among populations, we used data from microsatellite loci and selected populations that were distributed around the island at various distances from one another (see Figure 1).

## Results

Our analyses were done in three parts. First we evaluated how phenotypic characteristics were related to one another to inform our selection of characteristics for further study (i.e., to identify orthogonal characteristics unlikely to be developmentally or genetically linked and subsequently more likely to exhibit independent variation from one another; NB: selection of characteristics also depended on which were most likely targets of selection). Second, we determined whether the allometries of ornaments within populations were consistent with characteristics under sexual selection. Finally, we tested whether indices of sexual selection, natural selection (predation) or genetic differentiation accounted for population variation in selected phenotypic characteristics.

### Principal component analysis

The relationship between phenotypic characteristics was assessed using principal component analysis (PCA). PCAs generally revealed two independent axes of phenotypic variation, one relating to characteristics of size (PC1: body size, head crest area, dorsal fin area) and another relating to characteristics of colour (PC2/3: percentage area of the dorsal fin coloured and intensity of red of the dorsal fin, ∆^R^/_G_; Table 1). Mean and standard deviations for all characteristics are provided in Additional file 1: Table S1. Based on these results, we selected body size, head crest size, and dorsal fin redness as representative features to examine phenotypic differentiation among populations as a function of variation in selection (see ‘Population divergences in phenotype’ below).

**Table 1:** Principal component axes of five phenotypic characteristics of the Pacific leaping blenny

**Characteristic**	**PC1**	**PC2**	**PC3**	**PC4**	**PC5**
Males, *N*_individuals, populations_ = 94, 5					
Body size*	**.98**	.04	.04	-.18	.11
Head crest size*	**.94**	.08	-.03	.32	.01
Dorsal fin, area	**.98**	.09	.00	-.13	-.12
Dorsal fin, proportion coloured	-.14	.**78**	**.61**	.01	.00
Dorsal fin, "reddness"*	-.12	**.79**	**-.60**	-.02	.01
Eigenvalue	2.84	1.25	.74	.15	.03
Cumulative variance explained (%)	56. 7	81.6	96.4	99.4	100.0
Females, *N*_individuals, populations_ = 97, 5					
Body size*	**.92**	-.33	.09	-.16	
Dorsal fin, area	**.90**	-.39	.09	.16	
Dorsal fin, proportion coloured	**.61**	**.66**	.43	.01	
Dorsal fin, "reddness"*	**.71**	.36	**-.61**	.00	
Eigenvalue	2.54	.83	.58	.05	
Cumulative variance explained (%)	63.5	84.3	98.8	100.0	

Prominent loadings (>.50) are highlighted in bold. Characteristics highlighted by an asterisk were examined in population differentiation analyses (Tables 4 and 5).

### Allometry

In a power function of the size of a morphological feature, *y*, on overall body size, *x* (*y* = *ax*^*b*^) the allometric exponent, *b*, represents the proportional growth of that feature relative to an animal’s body size within a population. Ornaments under sexual selection are expected to exhibit positive exponents of 1.5 or greater [30]. In the same power function, the allometric elevation, *a,* reflects the overall size of a feature in respect to body size for a population. Sexual selection on the exponent would presumably lead to increases in elevation over evolutionary time as well (e.g., [31]).

As predicted for a sexually selected ornament, the allometric exponents of male head crest size and male dorsal fin size within a population were all greater than 1.5 (Table 2). We also compared these exponents with a control region—the ventral fin—to provide an additional benchmark of the extent to which head crest and dorsal fin exponents might reflect sexual selection and not some other phenomenon (see Methods). Again, consistent with ornaments subject to sexual selection, the computed 95% confidence intervals for the majority of male head crest and male dorsal fin exponents did not overlap the confidence intervals of exponents computed for the ventral fin control region. There were some exceptions. First, neither the head crest nor dorsal fin of males from the Talofofo population could be reliably identified as exhibiting exponents greater than the control ventral fin (Table 2). This population also had the lowest recorded sex ratio, which implies competition among males for females was low (see next section). Second, the dorsal fin of males from Umatac was also indistinguishable from the estimated exponent for the control ventral fin, but the exponent of the head crest for this population was the largest estimated exponent for any population and any morphological feature (Table 2).

**Table 2:** Allometric coefficients for head crest area, dorsal fin area and ventral fin area (a non-sexually selected control region)

	**Males**			**Females**		
**Population, character**	**N**	***a***_**elevation **_**(lower 95% CI, upper 95% CI)**	***b***_**exponent **_**(lower 95% CI, upper 95% CI)**	**N**	***a***_**elevation **_**(lower 95% CI, upper 95% CI)**	***b***_**exponent **_**(lower 95% CI, upper 95% CI)**
Pago						
Head crest area	25	1.13 (1.05, 1.20)	2.50 (1.96, 3.03)*		na	na
Dorsal fin area	25	2.70 (2.67, 2.73)	1.82 (1.63, 2.01)*	26	2.45 (2.41, 2.49)	1.43 (1.19, 1.67)
Ventral fin area	23	2.36 (2.33, 2.38)	1.25 (1.07, 1.43)	24	2.22 (2.18, 2.25)	1.23 (1.04, 1.43)
Taga'chang						
Head crest area	54	1.10 (1.04, 1.15)	2.24 (1.86, 2.62)*		na	na
Dorsal fin area	54	2.78 (2.76, 2.80)	1.53 (1.39, 1.67)*	53	2.41 (2.39, 2.43)	1.32 (1.19, 1.46)
Ventral fin area	41	2.41 (2.39, 2.43)	1.22 (1.08, 1.36)	36	2.23 (2.21, 2.25)	1.12 (.97, 1.27)
Talofofo						
Head crest area	44	1.27 (1.22, 1.32)	2.03 (1.50, 2.57)		na	na
Dorsal fin area	44	2.88 (2.86, 2.90)	1.59 (1.37, 1.81)	53	2.33 (2.31, 2.34)	1.49 (1.38, 1.60)
Ventral fin area	27	2.55 (2.52, 2.59)	1.49 (1.18, 1.81)	35	2.26 (2.23, 2.29)	1.31 (1.10, 1.51)
Umatic						
Head crest area	24	1.07 (.98, 1.16)	2.62 (2.14, 3.10)*		na	na
Dorsal fin area	24	2.73 (2.69, 2.77)	1.61 (1.39, 1.84)	28	2.39 (2.35, 2.43)	1.47 (1.27, 1.68)
Ventral fin area	24	2.40 (2.36, 2.44)	1.25 (1.04, 1.46)	28	2.30 (2.26, 2.34)	1.22 (.99, 1.46)
Adelup						
Head crest area	24	.85 (.78, .92)	2.14 (1.74, 2.55)*		na	na
Dorsal fin area	24	2.61 (2.58, 2.63)	1.71 (1.57, 1.85)*	25	2.41 (2.38, 2.43)	1.42 (1.20, 1.65)
Ventral fin area	20	2.29 (2.26, 2.32)	1.33 (1.16, 1.51)	23	2.27 (2.24, 2.30)	1.27 (1.02, 1.52)

NB: females lack a headcrest. Coefficients with 95% confidence intervals that do not overlap the confidence intervals of the ventral fin are highlight with an asterisk.

Allometric exponents for female dorsal fin area were also positive, but lower than 1.5 and had computed 95% confidence intervals that overlapped the confidence intervals of the ventral fin control region.

**Figure 2:**
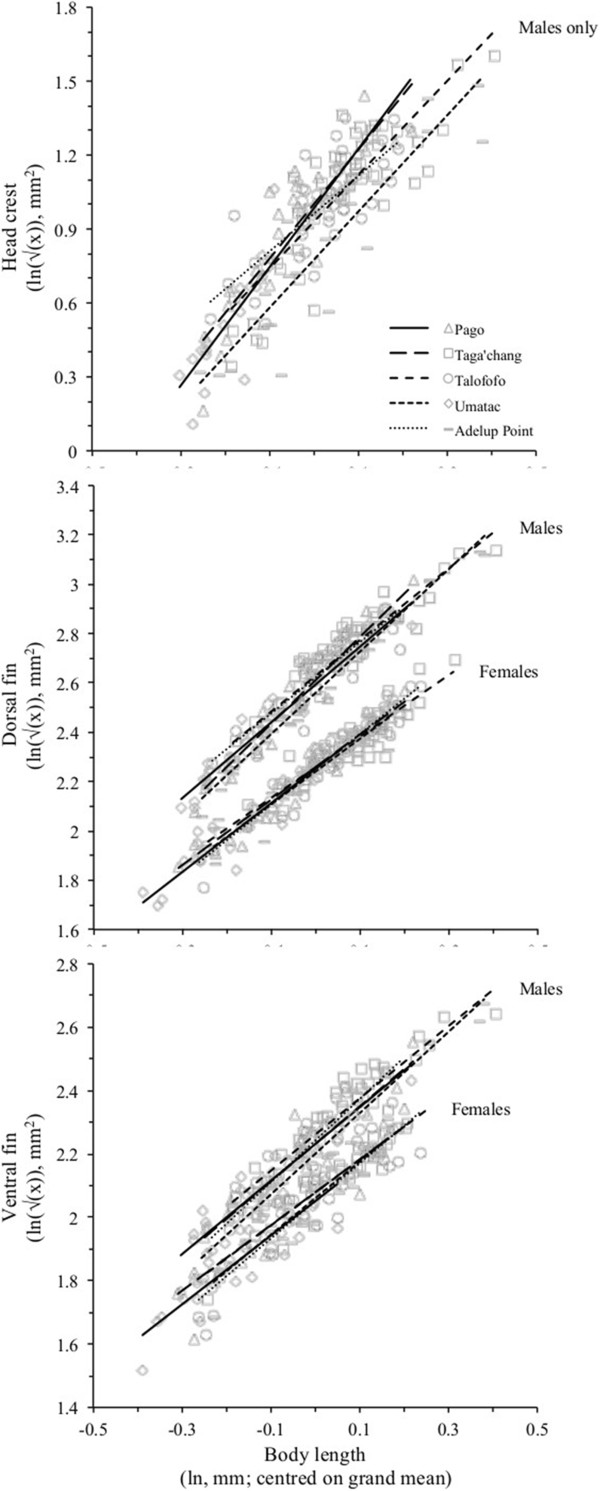
**The allometry of ornamentation in the Pacific leaping blenny.** Shown are natural-log data for the head crest, dorsal fin and ventral fin as a function of body length. The regression lines are the linear analogue of the allometric power function in which the allometric elevation corresponds to the intercept value, while the allometric exponent is represented by the regression slope (i.e., larger exponents result in steeper slopes and show the degree larger males invest disproportionally more in exaggerated ornamentation compared to smaller males). In order to properly estimate the allometric equation, all characteristics must be on the same scale. Crest and fin areas were therefore converted to a linear scale to match that of body length through a square-root transformation before then being natural-logged.

Comparison of the estimated elevations for head crest area, dorsal fin area and ventral fin area was not relevant because the size of these features were different (i.e., the head crest was substantially smaller than the dorsal and ventral fins, and the dorsal fin was larger than the ventral fin). However, comparisons can be made for the same feature between the sexes, and this revealed strong male-biased size dimorphism in both the dorsal and ventral fin (NB: these differences are independent of overall body size; Table 2; Figure 2).

Overall, male head crest area and dorsal fin area exhibited allometries consistent with sexually selected features, in both the magnitude of exponents and sexual dimorphism in elevations (Figure 2). The particularly large allometric exponents for male head crest area (Table 2; Figure 2) also reaffirmed our selection of this characteristic for the study of phenotypic differentiation among populations (see next section). In contrast, the allometry of female dorsal fin area could not be reliably distinguished from features that were not sexually selected (ventral fin area; Table 2; see also Figure 2).

### Population divergence in phenotype

We evaluated the extent and cause of divergence in phenotype among our five populations using a model selection approach. Models with the lowest AIC value were considered the best supported models, although any model within two units of these lowest models (ΔAIC ≤ 2.0) were also considered biologically viable models (see [32]). When the null model in model sets obtained compelling support (i.e., ΔAIC ≤ 2.0), we followed up with Mantel Tests to determine whether phenotypic variation among populations was correlated with genetic differentiation.

Overall, predation (specifically strikes to model blennies), sex ratio and the null model were generally ranked as the highest models accounting for phenotypic variation among populations in both sexes. However, the nature of relationships was not straightforward and only population differences in head crest allometry and the redness of the dorsal fin in males could be reasonably attributed to differences in selection. Genetic differentiation among populations as measured using *D*_est_ was extremely low (range: 0.0009 to 0.007; see Table 3). This result effectively excluded at the outset the possibility that neutral genetic drift among populations might account for any phenotypic divergence since there was virtually no genetic differentiation between populations. We detail the results for our three representative phenotypic characteristics below.

**Table 3:** **Pairwise *D***_**est **_**comparisons among the five sampled populations**

	**Pago**	**Taga'chang**	**Talofofo**	**Umatic**	**Adelup**
Pago	0	0.00158	0.00261	0.00654	0.00157
Taga'chang	-	0	0.00198	0.00088	0.00533
Talofofo	-	-	0	0.00584	0.00411
Umatic	-	-	-	0	0.00554
Adelup	-	-	-	-	0

#### Body size

**Figure 3:**
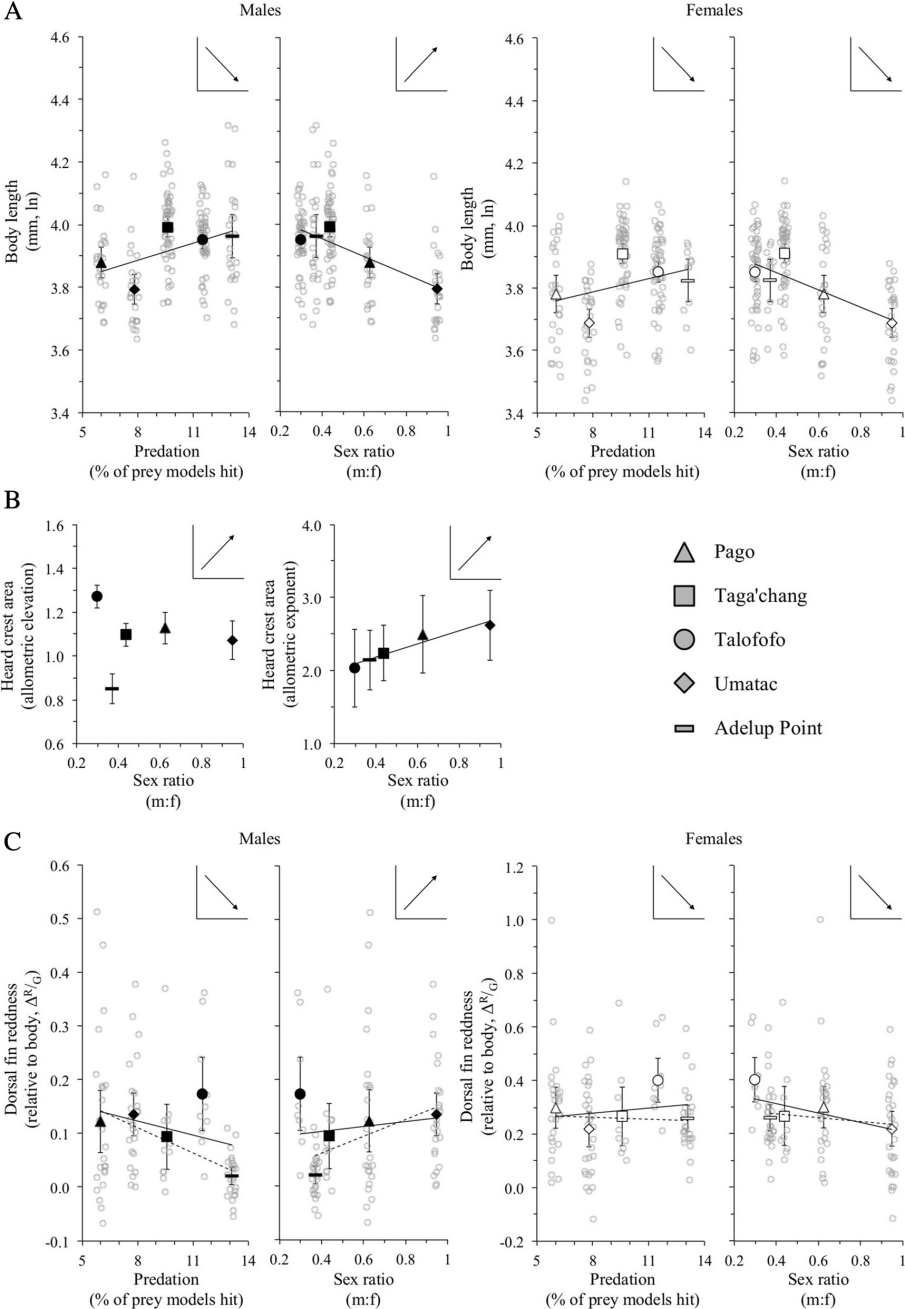
**Population variation in phenotype.** Shown is **(A)** adult body size, **(B)** the allometric parameters of the male head crest, and **(C)** the intensity of red of the dorsal fin (Δ^R^/_G_) as a function of predation (strikes to blenny models) and sex ratio. Grey, open circles are data points for individual fish, while black filled (male) and white filled (female) symbols are population means or allometric coefficients computed from RMA regressions reported in Table 2. Error bars correspond to 95% confidence intervals. Insets are predicted trends assuming predation selects against conspicuous characteristics, while sexual selection increases the conspicuousness of characteristics. For males, competition for mates was expected to increase with male biased sex ratios. For females, competition for mates was expected to decrease with male biased sex ratios. Solid trend lines are inclusive of all populations. Dashed trend lines exclude Talofofo (a population with an unusually red dorsal fin; C). Trend lines were computed based on parameter estimates from the best-supported models in Table 4 (those with ∆AIC ≤ 2.0).

The highest ranked model for both male and female body size was one that predicted population differences in size as a function of differences in predation pressure among populations (Table 4; NB: predation was measured as the number of attacks on blenny models placed in the environment for three days; there was virtually no sign of attack on controls, which were conspicuous, non-food related objects over the same period (see Methods and Additional file 2: Figure S1 for details) suggesting that attacks to the blenny models were a reasonable reflection of blenny-specific predation). Sex ratio was another plausible predictor of male size (∆AIC_c_ < 2.0; Table 4A), while the null model was also ranked highly for female size (∆AIC_c_ < 2.0; Table 4B; and to some extent male size as well: ∆AIC_c_ ~ 2.0; Table 4A). But the effects of all of these models were not especially compelling: effect sizes were all small and often counter-intuitive in their direction of influence. For example, we might expect that predation leads to a general decrease in body size, but the direction of influence highlighted by effect sizes and visual inspection of plots suggested a positive relationship between predation and body size (if a relationship existed at all; see Figure 3A). Populations under high predation pressure could exhibit increased mean body size if predation resulted in high juvenile mortality (i.e., predators preferentially preyed on juveniles over adults or if predators were gape-limited and unable to consume large individuals; e.g., [33]). However, predation was explicitly measured on adults (i.e., the number of strikes to adult sized prey models), so this explanation is unlikely. Similarly, while the null model ranked comparatively high for both sexes, subsequent Mantel Tests revealed little relationship with neutral genetic differentiation among populations (*r* = −0.05 to −0.11). This was not surprising given the almost complete lack of genetic differentiation among populations (Table 3).

**Table 4:** Predictors of (A) male and (B) female population differentiation in three representative phenotypic characteristics

				**Selection effect size**	
**Model**	**AIC**_**c**_	**∆AIC**	**AIC**_**w**_	***r***_**sexual**_	***r***_**natural**_
**A. Males**					
Body size*					
*N*_individuals, populations_ = 171, 5					
Predator strikes	−209.81	.00	.32	nil	.11
Sex ratio	−209.37	.44	.26	.04	nil
Sex ratio + predator strikes	−209.28	.53	.25	-.18	-.01
Null	−207.59	2.22	.11		
Intrasexual density + predator strikes	−205.08	4.73	.03		
Total density + predator strikes	−203.55	6.26	.01		
Intrasexual density	−202.40	7.40	.01		
Sex ratio + flight distance	−201.89	7.92	.01		
Flight distance	−201.48	8.33	.01		
Total density	−199.04	10.77	.00		
Intrasexual density + flight distance	−196.36	13.44	.00		
Total density + flight distance	−193.28	16.52	.00		
Head crest, allometric exponent					
*N*_populations_ = 5					
Sex ratio	−1.65	.00	.93	.96	nil
Null	4.31	5.96	.05		
Intrasexual density	6.47	8.13	.02		
Total density	10.86	12.51	.00		
Head crest, allometric elevation					
*N*_populations_ = 5					
Null	-.45	.00	.89	Nil	nil
Total density	5.38	5.83	.05		
Intrasexual density	6.16	6.61	.03		
Sex ratio	6.20	6.65	.03		
Dorsal fin "reddness"*					
*N*_individuals, populations_ = 94, 5					
Predator strikes	−165.82	.00	.44	nil	-.10
Null	−165.47	.35	.37	nil	nil
Sex ratio + predator strikes	−161.80	4.03	.06		
Intrasexual density + predator strikes	−161.58	4.24	.05		
Sex ratio	−160.90	4.92	.04		
Intrasexual density	−159.00	6.82	.01		
Flight distance	−158.19	7.63	.01		
Total density + predator strikes	−156.49	9.33	.00		
Total density	−156.04	9.79	.00		
Sex ratio + flight distance	−153.91	11.91	.00		
Intrasexual density + flight distance	−151.88	13.94	.00		
Total density + flight distance	−149.13	16.69	.00		
Dorsal fin "reddness", without Talofofo*					
*N*_individuals, populations_ = 84, 4					
Predator strikes	−151.15	.00	.65	nil	-.32
Null	−148.08	3.07	.14		
Sex ratio + predator strikes	−146.73	4.42	.07		
Intrasexual density + predator strikes	−145.95	5.20	.05		
Sex ratio	−145.66	5.50	.04		
Intrasexual density	−145.62	5.54	.04		
Total density + predator strikes	−140.60	10.55	.00		
Flight distance	−140.52	10.63	.00		
Total density	−138.90	12.25	.00		
Sex ratio + flight distance	−137.59	13.56	.00		
Intrasexual density + flight distance	−137.36	13.79	.00		
Total density + flight distance	−132.15	19.01	.00		
**B. Females**					
Body size*					
*N*_individuals, populations_ = 185, 5					
Predator strikes	−225.21	.00	.46	nil	.03
Null	−223.87	1.34	.23	nil	nil
Flight distance	−221.91	3.30	.09		
Sex ratio + predator strikes	−221.01	4.20	.06		
Intrasexual density + predator strikes	−220.98	4.23	.06		
Sex ratio	−220.44	4.77	.04		
Total density + predator strikes	−220.40	4.81	.04		
Intrasexual density	−218.67	6.54	.02		
Total density	−216.11	9.10	.00		
Sex ratio + flight distance	−214.02	11.20	.00		
Intrasexual density + flight distance	−212.45	12.76	.00		
Total density + flight distance	−210.37	14.84	.00		
Dorsal fin "reddness"*					
*N*_individuals, populations_ = 97, 5					
Null	−110.51	.00	.37	nil	nil
Predator strikes	−110.29	.22	.33	nil	.05
Sex ratio	−108.97	1.55	.17	.28	nil
Sex ratio + predator strikes	−107.59	2.92	.09		
Flight distance	−103.88	6.64	.01		
Intrasexual density + predator strikes	−102.68	7.84	.01		
Intrasexual density	−102.61	7.91	.01		
Sex ratio + flight distance	−102.18	8.34	.01		
Total density + predator strikes	−101.29	9.23	.00		
Total density	−100.87	9.64	.00		
Intrasexual density + flight distance	−95.81	14.71	.00		
Total density + flight distance	−94.04	16.47	.00		
Dorsal fin "reddness", without Talofofo*					
*N*_individuals, populations_ = 87, 4					
Null	−97.38	.00	.56	nil	nil
Predator strikes	−96.32	1.06	.33	nil	-.01
Sex ratio	−92.40	4.98	.05		
Sex ratio + predator strikes	−91.15	6.23	.02		
Flight distance	−90.51	6.87	.02		
Intrasexual density	−89.42	7.96	.01		
Intrasexual density + predator strikes	−88.33	9.05	.01		
Total density	−87.37	10.01	.00		
Total density + predator strikes	−86.74	10.64	.00		
Sex ratio + flight distance	−85.54	11.84	.00		
Intrasexual density + flight distance	−83.78	13.61	.00		
Total density + flight distance	−80.59	16.80	.00		

Model support was evaluated using Akaike’s Information Criterion (AIC_c_). Effect sizes indicating the magnitude and direction of effects are reported for models receiving the most support (ΔAIC ≤ 2.0) and shown graphically in Figure 3. Models that included a random effect for population are highlighted with an asterisk.

In short, no selection model or neutral evolution model provided a convincing explanation for body size variation among populations.

#### Head crest allometry

We used the computed allometry elevations and exponents as population-level dependent variables (see Table 2). As male head crests are relatively small, they are difficult to identify from a distance and, as such, we assumed they would not increase susceptibility to predation independently of body size (i.e., only indexes of sexual selection were considered in models). Sex ratio was clearly the only compelling model explaining variation in the exponent of male head crest allometry, with a positive and very large effect size (*r* = 0.96; Table 4A). As competition for females increased among males in a population, the magnitude of the allometric exponent has increased across populations (Figure 3B), inferring that the exaggeration of the head crest is the product of sexual selection.

In contrast, no selection model provided a compelling explanation for the allometric elevation of the head crest (Table 4A). Here, only the null model obtained any support (as a comparison with the prominent relationship between the exponent and sex ratio, we provide the companion plot for elevation in Figure 3B). A subsequent Mantel Test failed to provide any evidence that differences in head crest elevation was instead a by-product of neutral genetic differentiation among populations (Table 5).

**Table 5:** Tests for neutral evolution

**Characteristic**	**Effect size, *r***	**β**_**intercept **_**(lower 95% CI, upper 95% CI)**	**β**_***D*est**_**(lower 95% CI, upper 95% CI)**	***P *value**
Males, *N*_populations_ = 5				
Body size	-.11	1.57 (.93, 2.20)	−224.80 (−379.64, −69.96)	.63
Dorsal fin, redness	.14	-.22 (−1.09, .66)	312.60 (98.18, 527.02)	.38
Head crest, elevation	.26	-.48 (−5.26, 4.30)	1747 (576.88, 2917.12)	.27
Females, *N*_populations_ = 5	-.05	1.61 (−.98, 2.23)	−221.40 (−374.67, −68.13)	.56
Body size	-.05	1.61 (−.98, 2.23)	−221.40 (−374.67, −68.13)	.56
Dorsal fin, redness	.13	-.24 (−.86, .38)	220.30 (68.99, 289.29)	.42

Mantel tests were used to determine whether supported null models in Table 3 reflected instances of neutral evolution.

#### Dorsal fin redness (Δ^R^/_G_)

The null model, predation pressure (strikes to blenny models) and to some extent sex ratio were plausible models accounting for population variation in the intensity of dorsal fin redness in both sexes (Table 4). Mantel Tests ruled out any relationship between population differentiation in fin colouration and neutral genetic differentiation among populations (Table 5). The next best supported models after the predation only and null models were sex ratio or a more complex model that incorporated both predation and sex ratio (males: ∆AIC_c_ ~ 4.0; females: ∆AIC_c_ < 2.0).In males, plots suggested that, in general, the intensity of dorsal fin redness decreased with predation pressure and increased with increasingly male-biased sex ratios (Figure 3C). In females, the relationship between dorsal fin redness and predation was unclear, but there was a negative trend evident between dorsal fin redness and sex ratio (i.e., female dorsal fins were more red in populations in which males were more scarce; Figure 3C). These general trends were broadly consistent with the prediction that sexual selection (via aggressive competition or mate choice) should increase the conspicuousness of ornaments, while predation should decrease the conspicuousness of ornaments.However, one population—Talofofo—was clearly an outlier in Figure 3C and the interpretation of relationships was highly dependent on whether this population was included or excluded: in males, its inclusion could have increased support for the null model; in females, its inclusion could have increased support for the sex ratio model. We repeated our model fittings with this population removed. Overall model ranks remained unchanged, but only the predator model received any credible support for either sex.

Taken together, the underlying causes of population variation in dorsal fin redness appeared complex, with some support for the negative influence of predation and—perhaps—the positive influence of sexual selection.

## Discussion

Before the implications of our findings can be fully appreciated, we need to first interpret the absence of genetic differentiation among populations. While ecological barriers isolate adult populations, the Pacific leaping blenny has a roughly one month pelagic larval phase (Platt and Ord, unpublished data). During this larval phase, larvae have the capacity to disperse several hundred kilometers around the island (Cooke GM, Schlub TE, Sherwin WB, Ord TJ: Understanding the spatial scale of genetic connectivity at sea: insights from a land fish and a meta-analysis. In review). Given the distance between our furthest populations was less than a hundred kilometers and that pelagic larval dispersal is a well-known cause of high genetic connectivity among marine fish (e.g. [34-36]; including pelagic marine fish populations around Guam [37]), the lack of genetic differentiation among populations of Pacific leaping blenny almost certainly reflects contemporary gene flow rather than the recent isolation of populations (see Cooke et al. for discussion).

This high gene flow among populations is significant because the general assumption has been that populations only phenotypically diverge in the absence of the homogenizing effects of gene flow [23,38]. This in turn predicts that features that might facilitate reproductive isolation are unlikely to evolve if members from different populations frequently mate with one another. This has generated considerable debate over the extent to which adaptive differentiation might occur in response to geographic gradients in selection (e.g., along habitat gradients) and the likelihood of sympatric speciation occurring in nature [39]. Yet there are compelling examples of both adaptive differentiation among parapatric populations and speciation occurring without geographic isolation [40-44]. The question is whether these are special cases or whether adaptive differentiation is more common in the presence of gene flow than currently assumed.

Our results suggest that phenotypic divergence among populations of the Pacific leaping blenny has resulted from a complex interaction of natural and sexual selection, and in the presence of high gene flow among populations (as evidenced by the absence of genetic differentiation among populations). Sexual selection measured by male-biased sex ratios was clearly associated with population divergence in the exaggeration of the male head crest, and has also potentially increased the conspicuousness of the dorsal fin colouration in males and females. In contrast, natural selection operating through predation appears to have dampened the conspicuousness of dorsal fin colouration in at least males.

The strong, positive correlation between male head crest allometry and sex ratio reflects an increase in ornament size as population sex ratios became increasingly skewed towards males. This implies that greater competition for females has led to an increase in the investment made by large males in the size of the head crest (selection on the allometric exponent). Males defend rock holes from other males and attempt to entice females to enter and spawn in these holes through an elaborate head-nodding display. The head crest is clearly visible during this display and presumably an additional feature used by females to assess the quality of males. Yet this divergence among populations in ornament size has occurred in the presence of high gene flow. Presumably, a large adult male from Talofofo would have more difficulty in mating with females at Umatac or Pago because he would be competing with more males and males with larger head crests (e.g., Figure 2). The question that follows is the extent differences in head crest allometry (and the color of male dorsal fins) are genetic or plastic in origin. A high rate of larval dispersal among populations, as suggested by our genetic data, implies ornaments are plastic. Such plasticity would likely be dependent on a population sex ratio that is in itself temporally variable (sex ratio will tend to reflect the sex of larvae that happen to settle at a given location). All populations might therefore share the same underlying (evolutionary) allometry in head crest expression, but the observed investment in large head crests by large males (leading to an exaggerated allometric exponent in a population) depends on the level of competition for mates in a given population at a given point in time. That is, the density of males dictates the extent to which male larvae settling at that location subsequently invest in the exaggeration of the head crest. This is not to say that plasticity breaks the allometric constraint associated with ornament development (small males will still be limited in the overall size of their head crests compared to larger males), rather that the extent males “choose” to invest to reach their maximum potential ornament size varies by population. This type of plasticity has been demonstrated for other sexually selected characteristics (e.g., call rate in crickets: [45,46]), but has generally not been considered in the context of the allometry of ornaments (but see [47]). If ornamentation is developmentally plastic, then ornament divergence among populations might still limit reproduction among adults from different populations, but genetic isolation would only occur if larval dispersal connecting populations was disrupted in some way. In this sense, plasticity in ornament expression has the potential to facilitate future reproductive isolation among populations if ocean currents or the nature of storm surges facilitating larval dispersal were to change around Guam (e.g., because of climate change). Future studies on the Pacific leaping blenny will need to assess the extent to which the size of the head crest is plastic and the implications of this for reproductive isolation among populations.

The population differences in the allometric exponent of head crest size, but not its allometric elevation, has other implications as well. Classically, evolutionary change in the allometric exponent of sexually selected features was believed to be constrained by the very developmental costs believed to produce the distinctive pattern of positive allometry in ornaments (see [48] and references cited therein). These costs should be less apparent in the allometric elevation of ornaments, which should subsequently be more evolutionary liable than the exponent [49,50]. This has been confirmed by selection experiments (e.g., [31,51]; see also studies reviewed by Bonduriansky [52]) and at least one phylogenetic comparative study that documented extremely slow evolutionary change in within-species exponents of sexually selected characteristics (which is consistent with the notion that the exponent is developmentally constrained in some way [48]).The findings of our study clearly differed from these past findings. The allometric exponent of a prominent ornament in the Pacific leaping blenny—the head crest—seemed to be highly reactive to changes in the intensity of sexual selection (specifically, sex ratio), whereas there was virtually no change in the allometric elevation of the ornament under the same conditions (Figure 3B). That is, plastic or genetic changes in allometric exponent in response to changes in sexual selection have not subsequently lead to an overall change in head crest size in a population (a change in head crest elevation): small males are still limited in their ability to produce larger head crests. This implies that allometric elevations are not plastic in the Pacific leaping blenny, and either the intensity of sexual selection has not been stable enough from generation to generation to induce adaptive change (e.g., sex ratios vary temporally) or that evolutionary change in allometric elevations are constrained in some way (which contradicts previous assumptions about the evolvability of the allometric elevation relative to the exponent).

Going beyond the study of ornament allometries, there have been few studies that have examined the potential trade-off between sexual selection and predation in the expression of ornaments and other sexual signals more generally. Of these studies, most have examined colour signals that are constantly exposed to both conspecifics and predators (e.g., body colouration and patterning; [9,53]). However, when an animal’s colour signal can be concealed from predators—for example, by restricting the colour signal to a part of the body only exposed during a social display to conspecifics [54]; this study—the influence of predation should be reduced. In the Pacific leaping blenny, the dorsal fin was only erected and visible during bouts of signaling [27]. In this sense, the impact of predation on the conspicuousness of the dorsal fin colouration should be low. Yet, our results suggested that the intensity of dorsal fin redness in males was potentially the target of predation (Figure 3C). In contrast, however, dorsal fin colouration was far more conspicuous in females (compare the range of Δ^R^/_G_ values in Figure 3C), but female dorsal fin colour did not appear to be influenced by predation. We suspect this reflects differences in the frequency of dorsal fin displays between the sexes, which were much higher in males than females (males: roughly one fin display every 15 minutes; females: roughly one fin display every hour; computed from the data archive of [27]). More generally, though, our results imply that any conspicuous behavior, if used frequently enough, can be the target of predation that can lead to measureable differences in ornament expression among populations.

There are, of course, other potential selection pressures that were not examined by our study and may also have contributed to population divergences in ornamentation or body size. For example, ornaments are only effective sexual signals if mates and rivals are able to distinguish those ornaments from habitat backgrounds. This was an implicit assumption in our study and has been confirmed by previous study for at least the dorsal fin [28]. Given that habitat backgrounds do tend to vary among locations [28], some of the variation in dorsal fin coloration could be attributed to selection for increased conspicuousness in local environments. Furthermore, red pigmentation in fish has often been shown to be carotenoid based and consequently diet dependent (e.g., [55]). Differences among populations in the availability of carontenoids in the environment are another source of potential variation that was not examined here. The availability of food resources more generally might also account for differences in body size among populations (Figure 3A).

## Conclusion

Our study documents phenotypic differentiation among populations that have apparently experienced extensive gene flow, and in phenotypic features important in reproduction (e.g., male head crests). There are a number of other studies that have reported similar findings in a range of taxa [56-61], suggesting that selection (or plasticity; this study) can frequently be strong enough to push populations to diverge in reproductively important features despite gene flow. In this respect, our findings are not entirely unexpected. However, our study is one of only a handful that has documented population divergence in ornamentation (e.g., see [9,62]). Furthermore, this divergence has been recent (and potentially plastic) because it has not yet led to changes in regions of the genome that are subject to the influence of genetic drift (i.e. microsatellite DNA). Our study may therefore represent an early snapshot of divergence in reproductively important features that could facilitate the formation of future reproductive barriers among populations under some circumstances.

Our study also highlights the importance of adopting an integrated approach for investigating the effects of selection. Phenotypes are invariably the product of a complex interaction of various positive and negative selective pressures, and these pressures need to be considered collectively if we are to fully understand the adaptive process. Furthermore, unless the history of connectivity among populations is also assessed, it is impossible to determine whether changes in phenotype have led to, or been caused by, neutral genetic differentiation among populations. In the first instance, changes in phenotype have the potential to act as barriers to gene flow through assortative mating, while in the latter instance, divergence may simply be the by-product of geographic isolation and may have little role in maintaining reproductive isolation on secondary contact. In the particular case of this land fish, the lack of genetic differentiation among populations suggests extensive dispersal among populations and the possibility that population divergences in ornamentation have a plastic origin. If so, such plasticity has important implications for how reproductive isolation might originate among populations and how the evolution of ornament allometries should be viewed.

## Methods

All work conducted as part of this study was approved by the University of New South Wales Animal Care and Ethics Committee and is described in protocol #11/36b, initially approved on the 10^th^ March 2011 and most recently reviewed on the 28^th^ February 2013.

The five populations of the Pacific leaping blenny (*A. arnoldorum*) were studied in 2009 and 2011 between April and August, which overlapped with the breeding season of the genus in the Northern hemisphere (e.g., compare with [63]). The 2009 field trip was for a separate study [27], but photographs of fish taken on that trip also contributed data on two populations in the current study (see next section). The bulk of the data collected, including demographic and predation studies, were from the 2011 field trip. The location of the five populations were Pago (13°25'39"N, 144°47'56"E), Taga’chang (13°24'16"N, 144°46'53"E; studied in both 2009 and 2011), Talofofo (13°20'34"N, 144°46'21"E; studied in both 2009 and 2011), Umatac (13°17'40"N, 144°39'29"E) and Adelup Point (13°28'52"N, 144°43'43"E; see Figure 1). We consider blennies found at these locations as separate populations because large beaches and other inhospitable habitat made adult dispersal among sites virtually impossible. Populations ranged in distance around the coastline of the island from 4 to 92 km from each other. At all locations, blennies were found in high abundance on moist rocks within the splash zone. For one population—Adelup Point—blennies were also found along a man-made concrete wall that extended out from the shoreline into open water. Photos of each location are given in Additional file 3: Figure S2.

### Quantifying phenotypic characteristics

A previous study on the Taga’chang and Talofofo populations [27] revealed that both sexes were likely to be the target of sexual selection: males were found to defend rock holes from other males and court females to lay eggs in these rock holes, while females were often aggressive to other females, small males and juveniles that ventured too close while feeding on the rocks. Social interactions were centered on dorsal fin displays (aggression and courtship), headnods (courtship in males) and physical displacements through shoving (aggression). For the current study, we measured several characteristics relevant to these social interactions: body size (presumably influential in aggressive disputes), head crest size (present only in males and likely sexually selected), dorsal fin size, the proportion of the dorsal fin coloured (defined as the area of pigmentation that clearly differed in colour from the posterior base of the dorsal fin), and the intensity of red of the dorsal fin for both sexes in each population (dorsal fin displays being an important and frequent feature in social communication).

Individuals were captured by hand using small aquarium nets and placed in opaque plastic holding containers in the shade until photographs were taken. A Canon EOS 7D digital SLR with an EFS 15–85 mm zoom lens (in 2011) or a Canon Rebel XSi digital SLR with a 55-mm macrolens (in 2009) was used for photography with images stored as high-resolution jpegs. Fish were photographed using standardized protocols in both years (e.g., see [27,28]). Briefly, fish were placed individually into zip-lock bags moistened with seawater. Full body photographs were then taken of each individual positioned side-on to the camera with the dorsal fin raised against a white standard background (X-Rite ColorChecker White Balance Card) and beside a ruler and a munsell colour chart (X-Rite mini Color-Checker; Additional file 4: Figure S3). Multiple photographs were taken of each individual to ensure adequate illumination and positioning of the fish for complete resolution of the length of the fish, full extension of the fins and colouration of the dorsal fins. Fish were then released back at the point of capture. NB: Estimates of maximum age of Pacific leaping blennies from otolith analysis suggest a generation time of roughly one year (ER Platt & TJ Ord, unpublished data). We were therefore confident that there was no overlap in individuals caught and photographed in 2009 and 2011.

#### Morphology

Morphological data was measured digitally by the first author (CLM) from photographs taken predominantly in 2011 (*N* = 190; 95 males and 95 females, from all five populations), with data supplemented from photographs of individuals from the Taga’chang and Talofofo populations in 2009 (N = 167, 90 females and 77 males). In total, 357 fish were measured, with a minimum of 24 males and 25 females per population.

In order to make measurements, photographs were first imported as JPEGS into ImageJ ver 1.42q (Rasband, 1997–2009; NIH). Measurements were made in pixels and then converted into millimetres using the ruler featured along side the fish in the photograph.

In addition to measuring dorsal fin characteristics and the head crest in males, measurements were also taken of body length (standard length, from the snout to the posterior end of the vertebral column) and the area of the ventral (anal) fin. The ventral fin was used as a control region for estimates of dorsal fin and head crest allometry (see ‘Statistical Analyses’ below). All measurements were taken three times from separate photographs of each individual and then averaged. Sample sizes for each morphological characteristic, for each population, are reported in Tables 1, 2 and 3.

#### Colour

We were specifically interested in population differences in dorsal fin colouration that reflected variation in sexual selection rather than variation in diet among populations that were unrelated to sexual selection (the intensity of red, especially in fin ornamentation in fish, often reflects the amount of carotenoids assimilated from food [60], and this could potentially vary by location independently of social factors). To this end, the intensity of red of the dorsal fin was measured relative to the amount of red exhibited in the body (variation in body colouration was more likely to reflect differences in diet and less likely to be a direct target of sexual selection; NB: although the body had very little red in its colouration generally, there was still variation in body colouration among populations – [28]).

These colour analyses were performed by the first author (CLM) and used the same photographs used for the morphological measurements (see previous section). The inCamera plug-in for Photoshop CS4 was applied to photographs to first standardise the colour and brightness of the image with the known RGB values of the munsell chart colour squares placed along side the fish in the photograph (see [28,64]). Following this calibration, the marquee tool in Photoshop was used to calculate the values of the red (R), green (G) and blue (B) colour channels of the coloured area of the dorsal fin and representative, similar sized areas of the body. Estimates of R, G and B are only informative relative to each other. We therefore followed previous studies [28,64] and used the ratio of R to G (NB: preliminary analysis showed that the specific choice of channels for this ratio were largely unimportant; see [28]). Colour estimates were only made from photographs with low within image lighting variance (photographs in which the standard deviations for the values of R, G and B across the colour squares of the munsell colour chart were less than 2.5). Colour was estimated for at least two photos per individual and the resulting ^R^/_G_ ratios were averaged. The average ^R^/_G_ for the body was then subtracted from the average ^R^/_G_ for the dorsal fin to obtain a relative measure of dorsal fin colouration to body colouration within populations, ∆^R^/_G_. Morgans and Ord [28] provide a detailed discussion of the assumptions of this type of colour analysis in relation to the range of light wavelengths reflected by natural objects and how colour might be viewed by different types of animals (see also [65]). In general, this type of analysis provides a broad measure of colour that covers the widest range of wavelengths likely to be used by conspecifics and potential predators of the blenny (birds, land crabs and lizards).

Initially, photographs from both 2009 and 2011 were analyzed. However, statistical tests showed mean population estimates of colour differed significantly between years. This could reflect differences in the cameras used [66], seasonal effects on fin colouration (photos in 2009 were taken at the start of the breeding season in April, while photographs in 2011 were taken in the latter half of the breeding season in August) or differences between years in the availability of resources affecting colouration (NB: there was no difference in morphological measurements between years). Rather than attempt to use all data, we focused our analyses of colour on data collected only from individuals surveyed in 2011, which resulted in smaller sample sizes for Taga’chang and Talofofo (see Tables 2 and 3), but eliminated the potential confound of year.

### Indexing sexual and natural selection

The demographic, behavioral and predation surveys described below were each performed in non-overlapping areas of the environment.

#### Number of mates and competitor density

The number of mates and the number of competitors in a population are variables that can be expected to influence the level of sexual selection operating on a population [67,68]. For instance, females in a population with many males are more likely to be selective of which males they choose to mate with, compared to females in a population where males are rarer. Population sex ratio has therefore been a frequent index of sexual selection, with female “choosiness” expected to increase with male-biased sex ratios [67-69]. Furthermore, when individuals within populations compete for access to resources affecting reproduction (food, shelters, nesting sites), the number of competitors for those resources will affect the level of aggressive competition in that population. Competitor number—typically individuals of the same sex—is another important index of sexual selection, which can operate independently of population sex ratio (e.g., males in a population with an even sex ratio might still experience intense competition for resources because the density of male competitors, reflecting overall population density, is high). We therefore used both sex ratio and intrasexual competitor density as separate, complementary metrics of the likely intensity of sexual selection operating in a population (see also [66]).

Sex ratio and competitor density were measured by setting out eight 50 cm by 50 cm quadrates for each population. These were placed at intervals of 10 paces along the shoreline and were pitched on rocks within the splash zone, just above the high tide line. The boundaries of the quadrates were defined by cotton twine fastened to the rock using nails and wire. Quadrates were monitored three times during high, low and mid tides over a one week period and at various times of day (depending on the times of the tides).

A single observer (CLM) surveyed each quadrate individually from a distance of approximately 10 meters. Trials began with an initial 10 minute acclimation period. This acclimation period reduced the effect of disturbance caused by the observer initially moving into the area. The length of the acclimation period was based on pilot observations that showed that blennies returned to normal activity within 10 minutes (normal activity included foraging and engaging in social interactions). Following this acclimation period, the observer recorded the number and sex of all Pacific leaping blennies found within the quadrate. Sex was determined by the presence or absence of a head crest in sexually mature individuals (fish larger than 4 cm). Small individuals (those less than 4 cm in length) were classified as juveniles (this distinction of sexual maturity by size was supported by dissections of fish revealing the development of the testes and eggs; ER Platt & TJ Ord, unpublished data).

To calculate population sex ratio, the ratio of the number of adult males to adult females was first averaged across the nine observations for a given quadrate, and then averaged again across all quadrates for a given population. This population sex ratio was assumed to be roughly equivalent to the population’s operational sex ratio (e.g., see [69]). The number of same-sex competitors in a population was calculated as the number of adult males or the number of adult females proportional to the area of the quadrate not submerged in water (although quadrates were generally above the high tideline, some tides reached higher levels than others and submerged some quadrates for some observations). To account for this, adult male or female number was expressed as the number of individuals per m^2^ and was first averaged across observations for a given quadrate, and then averaged again across quadrates to provide a population estimate.

#### Flight distance and prey models

Flight initiation distance (FID) is a common metric used to estimate the “fearfulness” of animals to potential predators (reviewed by [70]). The assumption is animals that frequently come into contact with predators are more fearful and subsequently take flight more quickly in the presence of a threat stimulus, compared to animals that rarely encounter predators [71,72]. In the context of the current study, populations experiencing high predation should retreat into rock holes or crevices sooner in the presence of a potential predation threat compared to populations experiencing low predation.

To measure flight distance, we followed the general protocol outlined by [73]. A single researcher (CLM) carefully positioned themselves approximately 7 m (13 paces) from a rock on which one or more adult Pacific leaping blenny were observed to be foraging (this start distance, *D*_start_, was kept as consistent as possible for all trials: lower and upper quartiles = 12–13 paces or 6.5-7 m, *N*_trials, populations_ = 297, 5). When there was more than one fish present, a specific individual was chosen as the subject. Individuals were approached in a straight line and at a regulated pace (the speed of approach was kept as consistent as possible for all trials). To prevent confounding effects, replications occurred in areas visually separated from previous test subjects. As the researcher approached, the total number of paces taken from the start position to the point the subject was observed to flee, *D*_flee_, was noted (first movement away from disturbance). Flight initiation distance was subsequently calculated as:

$$\mathrm{FID}=D_{\mathrm{start}}–D_{\mathrm{flee}}$$

Here, larger values of FID correspond to greater fearfulness.

This procedure was replicated for at least 20 different males and 20 different females for each population to compute an average sex-specific flight distance for a given population.

While flight distance is a useful, intuitive metric reflecting potential predation pressure on a population, there are a number of ecological and social variables that can influence when animals decide to flee from a potential predation threat (e.g., proximity and availability of refuges, proximity and number of conspecifics; see [70]). These variables have the potential to vary across populations independently of predation pressure. To provide a complementary, alternative estimate of predation, we also deployed highly realistic plasticine mimics of the Pacific leaping blenny and recorded the number of predator strikes to these prey models. The use of plasticine or clay models has been a successful method for estimating predation pressure in a variety of taxa (e.g., [54,74,75]), including this species of fish [28].

Models were made from silicone casts of eight euthanized blennies (four adult males and four adult females; individuals were euthanized following procedures outlined in the UNSW ACEC protocol 11/36B). Specimens were then frozen and set in liquid silicone (CopyFlex™ Culinart Inc.). Once cured, specimens were gently removed from the silicone to produce a highly durable flexible rubber cast that captured minute detail of the morphology of the fish. The models themselves were created using black, yellow and white plasticine (Colorific) blended by hand to match the natural colour of blennies in life (based on photographs; see Additional file 2: Figure S1 and [28] for colour analysis of models). This plasticine was pressed into the cast, along with a black cable tie inserted into the model to act as an anchor point. Fishing line was then used to fasten the models securely to rocks within the splash zone above the high tide water line (i.e. habitat frequented by the blennies; see Additional file 2: Figure S1). Note, models did not include a raised dorsal fin and subsequently only mimicked the size and body coloration of adult blennies. Our primary goal was to obtain a baseline estimate of predation independent of the colour of the dorsal fin. Control models consisted of uniform pink plasticine shaped in a ring embedded with a yellow cable tie for anchorage. Controls were designed to represent highly visible, novel objects (i.e., non-food related items; see [28]).

Models and controls were alternated along the shoreline at regular intervals separated by at least 1.5 meters. The experiment was conducted in two halves with stimuli positioned in areas of the environment that did not overlap. The integrity of stimuli was briefly checked daily (a ~30 minute period of disturbance) and evidence of predation was tallied after three days, following which all stimuli were removed from the environment.

On day three, stimuli were categorised based on the following criteria: 1) no marks; 2) single or multiple small nicks; 3) large punctures or nicks; 4) entire portions missing; or 5) only the anchor point remaining. Findings of Morgans and Ord [28] showed the most accurate estimate of predation was provided by category 4 because it was only found on model blennies and never on controls and was clearly the result of predatory attempts rather than scavenging animals. Category 5 was exempt from analysis as it probably resulted from wave action or human interference with the stimuli. The percentage of models exhibiting signs of predation was subsequently calculated as:

$$\%\;\mathrm{Predation}=100\left(\frac{N_{\mathrm{category}4}}{N_{\mathrm{category}1}+N_{\mathrm{category}2}+N_{\mathrm{category}3}+N_{\mathrm{catgory}4}}\right)$$

### Indexing neutral genetic differentiation among populations

To assess the extent of neutral genetic drift between populations, we genotyped 204 fish using microsatellite loci. Seventeen males and seventeen females were collected from each site. Fish were euthanized, and muscle tissue was dissected and preserved in 20% DMSO in a saturated NaCl_2_ solution. DNA was extracted using a DNeasy blood and tissue extraction kit (Qiagen). Twenty microsatellite loci were originally developed using 454 Next-generation sequencing technology and selected following the protocol of Gardner et al. [76] (microsatellite primers and their reaction conditions are described in Cooke GM, Schlub TE, Sherwin WB, Ord TJ: Understanding the spatial scale of genetic connectivity at sea: insights from a land fish and a meta-analysis. In review). Of these, only 16 markers were employed in this study. These markers were chosen because they were polymorphic (an average of 16 alleles per locus and observed heterozygosity 0.65 - 0.7) and because significant deviation from Hardy-Weinberg equilibrium across all loci and populations (assessed using Arlequin [[77]]) was only observed in a few loci and was not consistent across populations. As such, these markers were considered to be informative for detecting population structure and recent population divergences. Multiplexed PCR products using fluorescently labeled primers were run at the Australian Genome Research Facility (AGRF, http://www.agrf.org.au) on a 3730 × l sequencer and the electropherograms were analyzed and scored manually using GeneMapper version 4.1 (Applied Biosystems). As we are not attempting to analyze the microsatellite data within a population genetics framework, the 16 polymorphic loci were used only to estimate genetic differentiation between the studied populations. To do this, we calculated the diversity measure *D*_est_[78] in SMOGD[79]. *D*_est_, which is based on the unbiased estimator *D*[79] was chosen for this purpose as it is arguably a more accurate measure of differences in allelic diversity than traditional measures such as *G*_ST_ and *F*_ST_[80].

### Statistical analyses

All morphological characteristics were ln-transformed prior to analyses to improve the normality of distributions. Statistical analyses were performed using the R ver 2.15.1 (R Development Core Team, The R Foundation for Statistical Computing, Vienna, Austria) unless otherwise stated.

#### Principal component analysis

We performed a PCA using the “principal” function in the package “pscyh” ver 1.3.10 [81] on body size, head crest area, dorsal fin area, proportion of the dorsal fin area coloured, and the intensity of red of the dorsal fin (∆^R^/_G_) on each sex separately, across individuals from all populations. We also repeated the analyses on each sex for each population separately to confirm component loadings were consistent for all populations (results not reported).

#### Allometry

We first converted dorsal fin and head crest area into a linear measure through a square-root transformation, and then performed a reduced major axis regression on body length (see [82]). If these morphological features were the target of sexual selection, we expected their exponents to be greater than 1.5 and greater than exponents of a control region not under sexual selection (see next paragraph). Although we also expected the area of the dorsal fin coloured red and Δ^R^/_G_ to be the target of sexual selection, we did not assess allometry in these features because dorsal fin area and area coloured were strongly related to one another (see Results) and the interpretation of allometry coefficients for Δ^R^/_G_ was unclear.

When interpreting allometric exponents, it is important to consider that non-sexually selected characteristics can also exhibit positive allometries (an exponent greater than 1; reviewed by [49]). With this in mind, we followed Bonduriansky’s [52] recommendation of comparing the allometric coefficients of our putative sexually-selected ornaments to a morphological feature that would not be reasonably expected to be the target of sexual selection. We choose the area of the ventral fin as our allometric “control” region. The ventral fin is hidden from conspecific observers in this land fish and its size is subsequently not visible for assessment by mates or rivals. Furthermore, the ventral fin may exhibit some positive allometry because of the biomechanical demands of swimming [83], which would also be true for the dorsal fin and perhaps even the head crest as well (e.g., if it functioned as a rudder). That is, although the Pacific leaping blenny rarely, if ever, ventures into the water, the dorsal fin or head crest might still exhibit positive allometry, not because of contemporary sexual selection, but because of an evolutionary history of swimming in marine ancestors (e.g., historical natural selection on swimming performance). Allometry comparisons with reference to the ventral fin should therefore provide some indication as to the extent to which the dorsal fin and head crest size is solely attributed to sexual selection. We did not use the tail fin as our control region because this feature is heavily used in locomotion on land [26] and its allometry in this capacity has not been assessed.

Computing allometric elevations is not straightforward if allometric exponents differ (as they did in our study). In this situation, the relationship between the estimated elevation and exponent in allometric equations is often correlated and the direction of this correlation dependent on the body size value where allometric curves tend to overlap among populations. To properly compute elevation estimates, we centred our body size measure (body length) on the grand mean across all populations (e.g., [48]; see also [84] for clarification on when data should be centred on a grand mean or individual group means). Failing to properly centre data leads to an inherent inverse relationship between the allometric elevation and exponent, which effectively renders the elevation estimate biologically uninterpretable [85].

#### Assessing population divergence in phenotype

We examined whether selection or neutral evolution might account for population variation in body size (length), head crest size (area), and the intensity of red of the dorsal fin (∆^R^/_G_) using a model selection approach. These features were selected following PCA and allometric analyses because they were associated with several other characteristics (and therefore representative) or were likely the product of selection. To assess the potential causes of population differentiation in these features, a range of biologically plausible statistical models were formulated and evaluated relative to each other using Akaike’s Information Criterion, with a correction for small sample size (AIC_c_). The model with the lowest AIC_c_ value was considered to be the best-supported model. By convention, however, any model within two AIC_c_ units of this lowest value (∆AIC_c_ ≤ 2) is considered equally plausible [32]. We also calculated model weights (AIC_w_) to provide a measure of the overall level of support for a given model relative to all other models considered [86]. Values ranged from 1.0, representing exclusive support for a given model, to 0.0, representing virtually no support for a given model.

Some models included only one index measure, while other models included a combination of one measure of sexual selection (either sex ratio or competitor density) and one measure of natural selection (either flight distance or predator strikes). It was not possible to formulate a model specifically for neutral genetic differentiation. This was because models assumed correlations between the means of the phenotypic and index measures, whereas the appropriate test for neutral genetic differentiation was one testing a correlation in the *distance* between phenotypic and index means—i.e., phenotypic distance in population means increases with the genetic distance of populations. Instead, we developed a null model that included no predictor variable. Instances where this null model was among the best-supported for a given model set (∆AIC_c_ ≤ 2) implied that population variance in mean phenotype was essentially random relative to all the index measures tested. We subsequently performed a Mantel Test to determine whether it was the level of neutral genetic differentiation among populations—indicative of genetic drift—that accounted for differences in population phenotypes (see below).

For body size and the intensity of red of the dorsal fin ∆^R^/_G_, we used random regression models in which the index measures were fitted as fixed effects with a random effect for population identity. These models were fit to the data using the ‘lmer’ package ver 0.999999-0 implemented in R [87]. For allometric elevations and exponents of head crest size, models were population level regressions and only included index measures as fixed effects. These models were fit to the data using the ‘lm’ function in the core package of R. Mantel tests were performed using IBDWS ver 3.23 [88], with p-values estimated using a permutation procedure of 10,000 random shuffles of the phenotypic matrix [80]. Phenotypic distances among populations were computed as standardized mean differences, or Cohen’s *d* (the difference between the mean values of two populations, divided by their pooled standard deviation; [87]). Genetic distances between populations were computed as *D*_est_ and specified as the predictor variable.

Last, we computed effect sizes in the form of *r*-values for all models that were well supported in model sets and for any subsequent Mantel Tests performed.

## Availability of Supporting Data

All data from this publication have been archived in the Dryad repository (doi 10.5061/dryad.r92b0) at http://datadryad.org[89].

## Competing interests

The authors have no competing interests.

## Authors’ contributions

CLM and TJO designed the study, conducted the analyses, and wrote the paper. CLM performed the fieldwork. GMC and TJO collected tissues samples for molecular work. GMC performed all molecular analyses and contributed to writing the paper. All authors approved the final version of the manuscript.

## Supplementary Material

Additional file 1: Table S1 Means and standard deviations (SD) of phenotypic characteristics measured in this study by population. Click here for file

Additional file 2: Figure S1 Stimuli used to measure predation. Shown are representative examples of an adult male blenny, the plasticine blenny model and the control in the typical (rocky) habitat of the Pacific leaping blenny. Click here for file

Additional file 3: Figure S2 Habitat of the Pacific leaping blenny. Shown are photographs of typical rocky habitat frequented by land blennies at the five sites surveyed (see Figure 1). Click here for file

Additional file 4: Figure S3 How phenotypic characteristics were quantified. Morphological and colour measurements were obtained by placing an adult fish along side a ruler and munsel colour palette. Areas highlighted in yellow correspond to areas of the photograph that were used to quantify colour. Click here for file
