# Supplementary material for: How populations differentiate despite gene flow: sexual and natural selection drive phenotypic divergence within a land fish, the Pacific leaping blenny
Source: BMC Evol Biol. 2014 May 6;14:97. doi: 10.1186/1471-2148-14-97 (PMC4055934; doi:10.1186/1471-2148-14-97)
Supplement: Additional file 1: Table S1 — Means and standard deviations (SD) of phenotypic characteristics measured in this study by population. [file 1471-2148-14-97-S1.doc]

Table S1 Means and standard deviations (SD) of phenotypic characteristics measured in this study by population

| **Characteristic** | **Pago** | | | **Taga’chang** | | | **Talofofo** | | | **Umatic** | | | **Adelup** | | |
| --- | --- | --- | --- | --- | --- | --- | --- | --- | --- | --- | --- | --- | --- | --- | --- |
| Males | **N** | Mean | SD | **N** | Mean | SD | **N** | Mean | SD | **N** | Mean | SD | **N** | Mean | SD |
| Body size (mm) | 25 | 48.75 | 6.32 | 54 | 54.91 | 6.85 | 45 | 52.29 | 5.12 | 24 | 44.75 | 5.81 | 24 | 53.35 | 9.52 |
| Head crest size (mm^2^) | 25 | 6.80 | 3.96 | 54 | 8.99 | 4.00 | 45 | 7.81 | 3.35 | 24 | 4.40 | 3.11 | 24 | 6.71 | 4.98 |
| Dorsal fin, area (mm^2^) | 25 | 169.65 | 86.05 | 54 | 249.21 | 92.26 | 45 | 204.14 | 59.34 | 24 | 123.68 | 53.78 | 24 | 215.12 | 131.84 |
| Dorsal fin, proportion coloured (%) | 25 | 0.14 | 0.05 | 54 | 0.14 | 0.03 | 45 | 0.15 | 0.05 | 24 | 0.17 | 0.04 | 24 | 0.11 | 0.04 |
| Dorsal fin, "reddness" (Δ^R^/G) | 25 | 0.12 | 0.15 | 11 | 0.09 | 0.10 | 10 | 0.173 | 0.11 | 24 | 0.13 | 0.10 | 24 | 0.02 | 0.04 |
| Females |  |  |  |  |  |  |  |  |  |  |  |  |  |  |  |
| Body size (mm) | 26 | 44.33 | 6.86 | 45 | 50.19 | 5.45 | 53 | 47.37 | 5.46 | 28 | 40.21 | 4.82 | 25 | 45.49 | 5.23 |
| Dorsal fin, area (mm^2^) | 26 | 89.75 | 39.51 | 45 | 116.42 | 33.14 | 53 | 101.92 | 32.76 | 28 | 65.13 | 22.23 | 25 | 89.27 | 27.55 |
| Dorsal fin, proportion coloured (%) | 26 | 0.21 | 0.03 | 45 | 0.21 | 0.08 | 53 | 0.22 | 0.06 | 28 | 0.19 | 0.04 | 25 | 0.21 | 0.05 |
| Dorsal fin, "reddness" (Δ^R^/G) | 26 | 0.30 | 0.07 | 10 | 0.27 | 0.18 | 10 | 0.40 | 0.13 | 28 | 0.23 | 0.17 | 25 | 0.26 | 0.12 |
