# Supplementary figures and images for: How populations differentiate despite gene flow: sexual and natural selection drive phenotypic divergence within a land fish, the Pacific leaping blenny
Source: BMC Evol Biol. 2014 May 6;14:97. doi: 10.1186/1471-2148-14-97 (PMC4055934; doi:10.1186/1471-2148-14-97)

Live animal

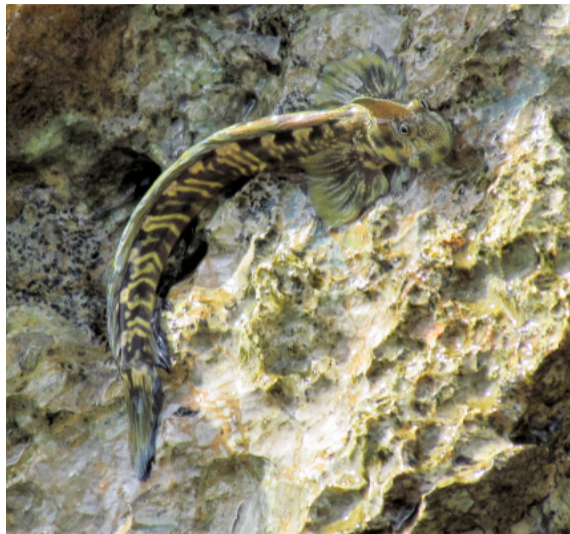

Model blenny

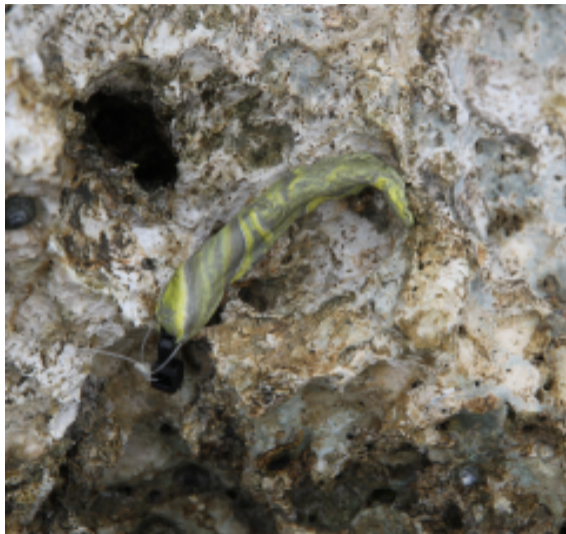

Control

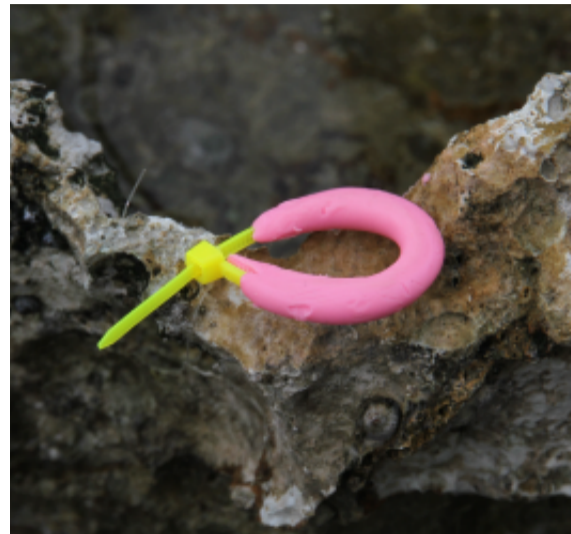

Supplement: Additional file 2: Figure S1 — Stimuli used to measure predation. Shown are representative examples of an adult male blenny, the plasticine blenny model and the control in the typical (rocky) habitat of the Pacific leaping blenny. [file 1471-2148-14-97-S2.pdf]

Guam

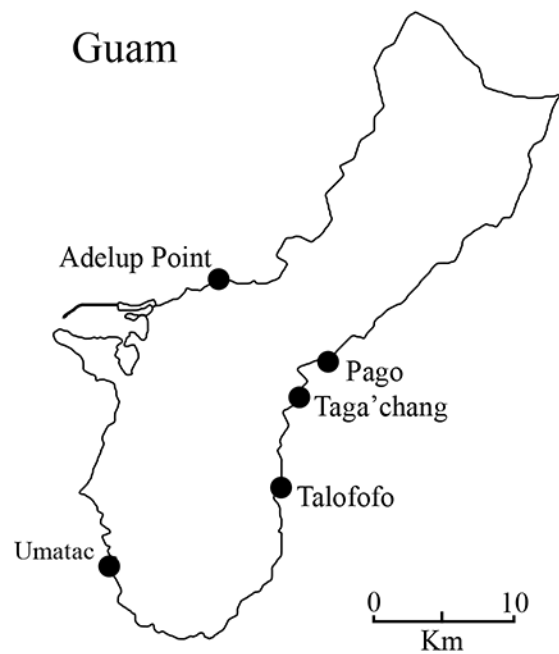

Pago

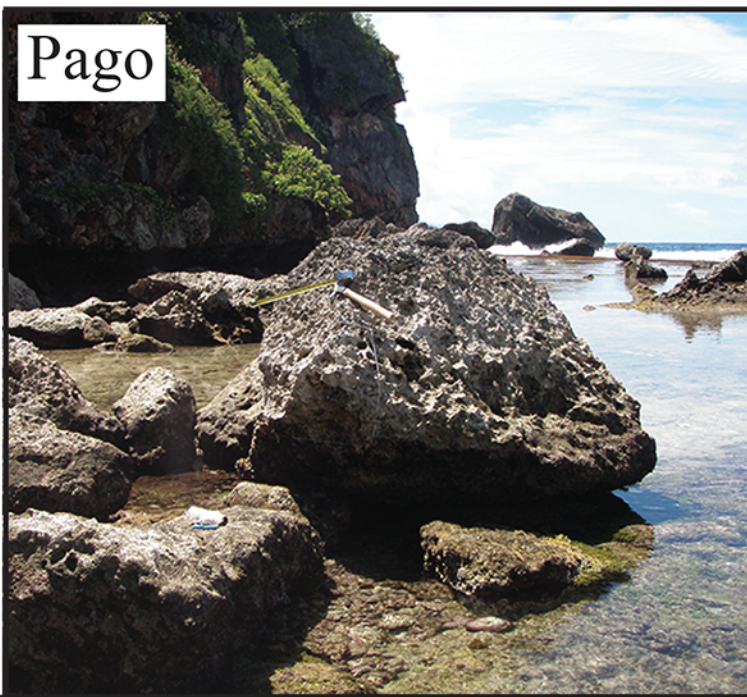

Taga'chang

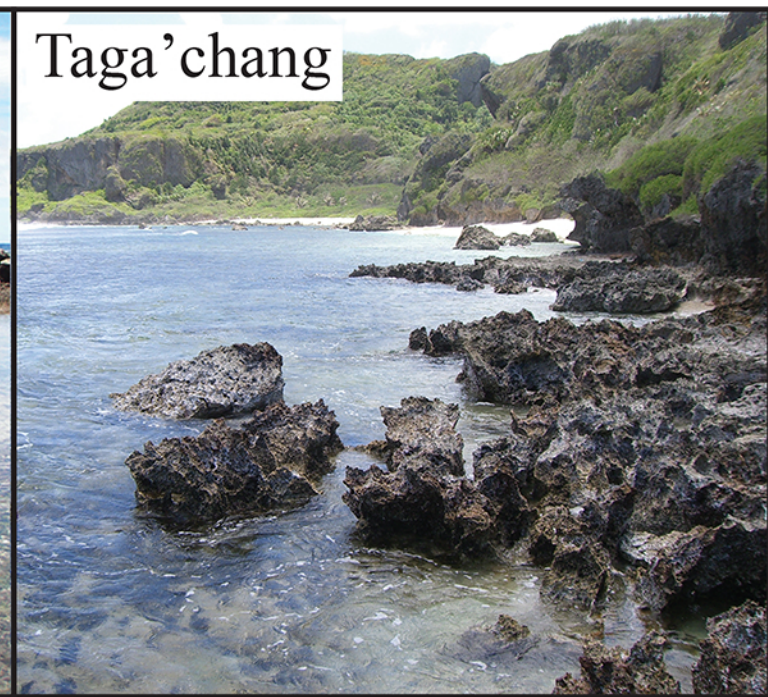

Talofoyo

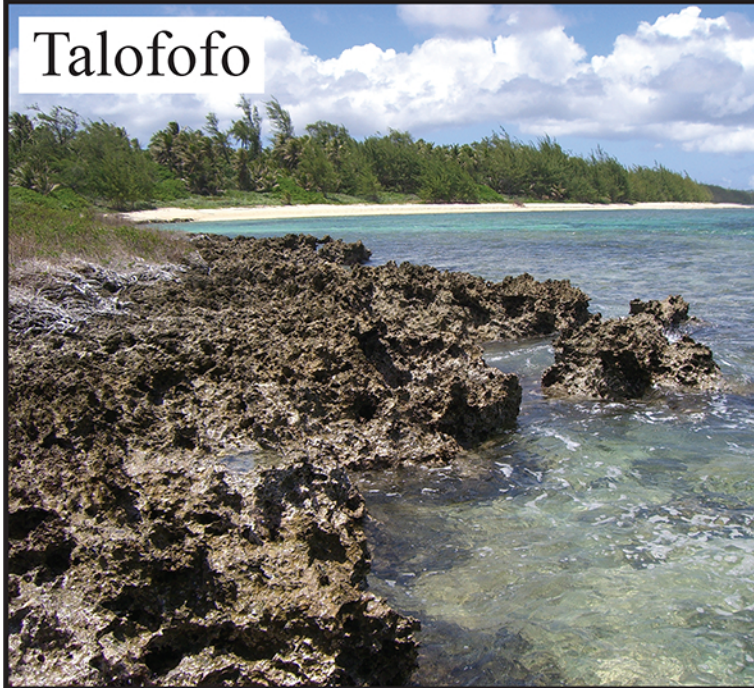

Umatac

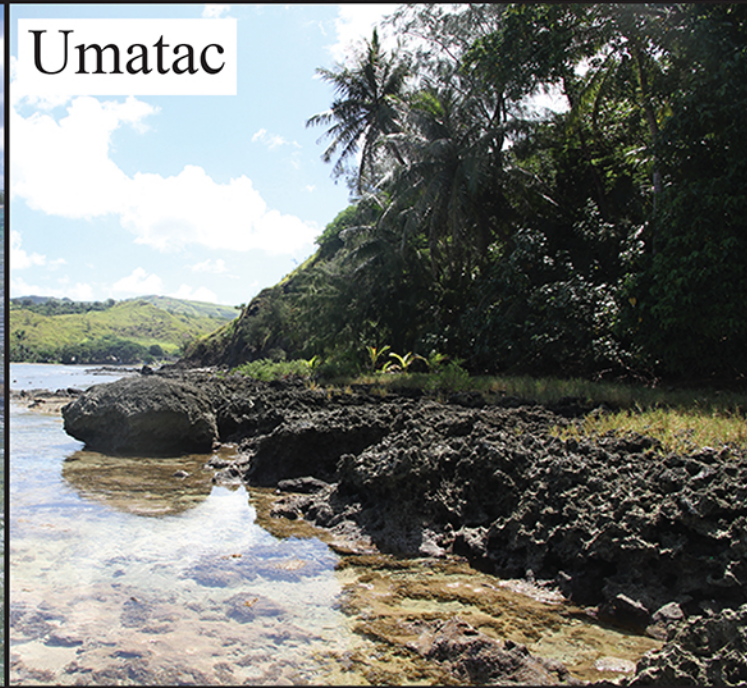

Adelup

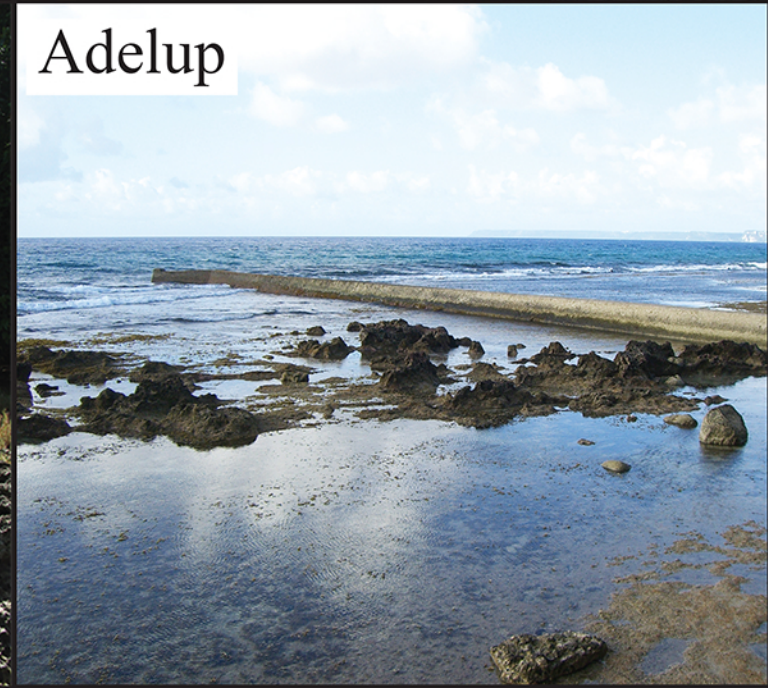

Supplement: Additional file 3: Figure S2 — Habitat of the Pacific leaping blenny. Shown are photographs of typical rocky habitat frequented by land blennies at the five sites surveyed (see Figure 1). [file 1471-2148-14-97-S3.pdf]

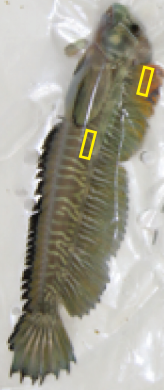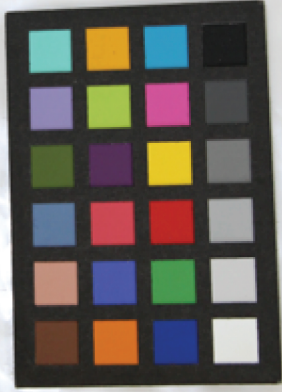

TC13

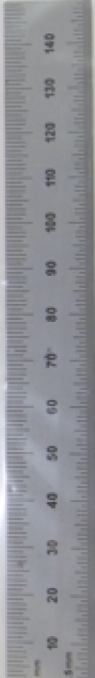

Supplement: Additional file 4: Figure S3 — How phenotypic characteristics were quantified. Morphological and colour measurements were obtained by placing an adult fish along side a ruler and munsel colour palette. Areas highlighted in yellow correspond to areas of the photograph that were used to quantify colour. [file 1471-2148-14-97-S4.pdf]
